# Disease-Modifying Drugs Reduce Cortical Lesion Accumulation and Atrophy Progression in Relapsing-Remitting Multiple Sclerosis: Results from a 48-Month Extension Study

**DOI:** 10.1155/2015/369348

**Published:** 2015-02-23

**Authors:** Francesca Rinaldi, Paola Perini, Matteo Atzori, Alice Favaretto, Dario Seppi, Paolo Gallo

**Affiliations:** Multiple Sclerosis Centre, Department of Neuroscience SNPSRR, University Hospital of Padova, Via Giustiniani 5, 35128 Padova, Italy

## Abstract

Cortical lesions (CLs) and atrophy are pivotal in multiple sclerosis (MS) pathology. This study determined the effect of disease modifying drugs (DMDs) on CL development and cortical atrophy progression in patients with relapsing-remitting MS (RRMS) over 48 months. Patients (*n* = 165) were randomized to sc IFN *β*-1a 44 *μ*g, im IFN *β*-1a 30 *μ*g, or glatiramer acetate 20 mg. The reference population comprised 50 DMD-untreated patients with RRMS. After 24 months, 43 of the untreated patients switched to DMDs. The four groups of patients were followed up for an additional 24 months. At 48 months the mean standard deviation number of new CLs was significantly lower in patients treated with sc IFN *β*-1a (1.4 ± 1.0, range 0–5) compared with im IFN *β*-1a (2.3 ± 1.3, range 0–6, *P* = 0.004) and glatiramer acetate (2.2 ± 1.5, range 0–7, *P* = 0.03). Significant reductions in CL accumulation and new white matter and gadolinium-enhancing lesions were also observed in the 43 patients who switched to DMDs after 24 months, compared with the 24 months of no treatment. Concluding, this study confirms that DMDs significantly reduce CL development and cortical atrophy progression compared with no treatment.

## 1. Introduction

The cortical pathology of multiple sclerosis (MS) is characterized by the accumulation of cortical lesions (CLs) [1–3] and a progressive thinning of the cortex [4–6]. CLs and gray matter atrophy can be detected with magnetic resonance imaging (MRI) at clinical onset [4], and, in some cases, precede the appearance of white matter lesions [7]. Increasing evidence suggests that CLs play a pivotal role in the physical and cognitive decline observed in patients with MS [8, 9]. In a previous 24-month Phase IV, randomized, longitudinal study we investigated the effects of DMDs on the development of CLs and atrophy in patients with RRMS [10]. Patients were randomized to receive subcutaneous (sc) interferon beta-1a (IFN *β*-1a; 44 *μ*g three times weekly), intramuscular (im) IFN *β*-1a (30 *μ*g weekly), or glatiramer acetate (20 mg daily) and were compared with untreated patients. Despite the older age, the apparently milder disease evolution, and the lower cortical lesion load of the untreated group (whose baseline demographic and clinical characteristics are described in detail in the original publication) [10], each regimen was found to delay the development of new CLs (*P* < 0.001). Moreover, a significant lower decrease in gray matter fraction was observed in DMD-treated patients compared with untreated patients (*P* = 0.023). Here we report a 24-month extension study that analyzed the long-term effects of DMDs on cortical pathology and evolution in patients who, after 24 months of no treatment, began active treatment.

## 2. Materials and Methods

### 2.1. Patients and Treatment

**Figure 1:**
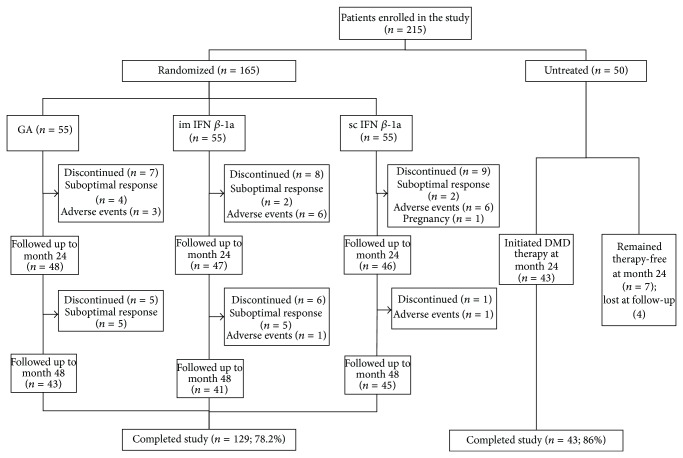
Patient enrollment, disposition, and follow-up. DMD: disease-modifying drug, GA: glatiramer acetate, IFN: interferon, im: intramuscular, and sc: subcutaneous.

The core study has been described previously [10]. Briefly, from January 1, 2007, to June 30, 2008, 215 patients with RRMS were enrolled in the study (Figure 1). Inclusion criteria were a diagnosis of RRMS according to the McDonald/Polman diagnostic criteria [11, 12], age 18–55 years, and an Expanded Disability Status Scale (EDSS) score of ≤5.0. Patients previously treated with immunosuppressive drugs were excluded. One hundred and sixty-five patients were assigned randomly (1 : 1 : 1) to receive sc IFN *β*-1a (44 *μ*g three times weekly), im IFN *β*-1a (30 *μ*g once weekly), or sc glatiramer acetate (20 mg once daily), while 50 patients with RRMS remained untreated. This untreated population was not randomized and reasons for not treating included both physician- and patient-driven factors, such as a relatively benign clinical course (low relapse rate and low WM lesion load on repeated MRI scans), needle phobia, female patients who were pregnant or trying for pregnancy, and interruption of previous therapy attempts with DMDs owing to persistent adverse effects or poor treatment adherence (in this case, a wash-out period of 3 months from therapy interruption was required before study entry), and philosophic or religious beliefs. Clinical disability was assessed every 6 months using the EDSS. MRI was performed at baseline and at months 12, 24, and 48 in all patients and in cases of relapse. In this study, a suboptimal DMD response was defined as the occurrence of a relapse determining an increase in disability of one EDSS point or the persistence of disease activity at MRI. After the first 24 months, since July 1 to December 31, 2010, a first-line therapy was initiated in 43/50 patients of the reference population. Given the low number of patients, therapy allocation was not randomized in this group but based on physician choice. Hence, 17 were treated with sc IFN *β*-1a, 12 with im IFN *β*-1a, and 14 with glatiramer acetate. These rather equally balanced patients were considered a unique group in the subsequent statistical analysis. The study was approved by the local Ethics Committee (HREC name: D. Piovan) and a written informed consent was obtained from all the patients.

### 2.2. MRI Methods

MRI procedure in the extension period was previously described [10]. Briefly, images were acquired using a 1.5-Tesla scanner (Achieva, Philips Medical Systems, Best, Netherlands) with a 33 mT/m power gradient and a 16-channel head coil. No major hardware upgrades occurred during the study. The following images were acquired for each patient: double-inversion recovery, fast fluid-attenuated inversion recovery, three-dimensional fast field echo, and postcontrast T1-weighted spin echo. Patients were carefully repositioned according to published guidelines for serial MRI studies of MS [13, 14]. All imaging was carried out at a single imaging center, and all images were assessed by the consensus of two experienced observers. The number of CLs, T2 hyperintense white matter lesion volume, gray matter fraction, and percentage change in gray matter volume between baseline and month 24 scans were calculated. The number of gadolinium-enhancing (Gd+) lesions was measured on postcontrast Gd+ T1-weighted images.

### 2.3. Statistical Methods

The Wilcoxon test was used to compare the 24- and 48-month time-point data (ARR, EDSS, MRI outcomes). The Huynh-Feldt epsilon correction method [13] was used to correct for interaction between groups and time. The Bonferroni correction was applied for between-group comparisons, during the first 24 months. A *P* value <0.05 was considered statistically significant. Stata 12.1 software (StataCorp, College Station, TX, USA) was used for all analyses.

## 3. Results

### 3.1. Patient Enrollment, Randomization, and Follow-Up

Of the 165 patients originally randomized to receive first-line DMDs, 129 (78.2%) completed the full 48 months of follow-up remaining on the originally allocated therapy, while 24 (14.5%) switched treatment during the first 24 months and 12 (7.3%) during the second 24 months of therapy (Figure 1). Since the majority of these patients were switched to second-line immunosuppressive therapies (e.g., natalizumab, fingolimod, cyclophosphamide, or mitoxantrone), these patients were omitted from the present analysis. Of the patients in the reference population (initially untreated; *n* = 50), 43 (86.0%) began treatment after the first 24 months. On the light of the results of the previous 24-month study and considering the low number of patients, treatment allocation was not randomized but mainly based on physician choice. As mentioned above, 17 patients initiated therapy with sc IFN *β*-1a, 12 with im IFN *β*-1a, and 14 with glatiramer acetate. This group was analyzed as a whole since the low number of patients in each subgroup did not allow a comparison between treatments. Four out of the seven patients that decided to remain therapy-free were lost during the follow-up; thus this group could not be used for statistical analyses.

### 3.2. Effect of DMD Treatment on New CLs, White Matter Lesions, Gd**+** Lesions, and Relapses

**Figure 2:**
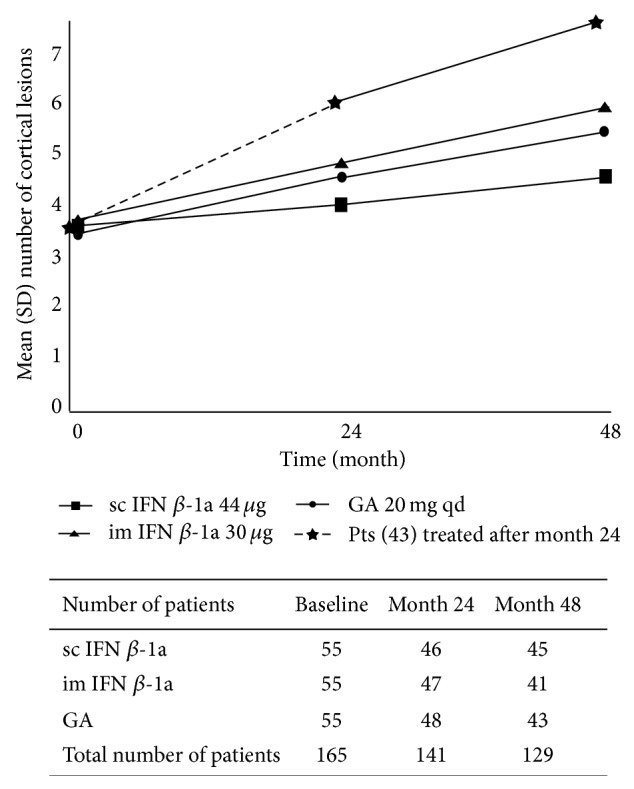
Mean number of total cortical lesions at baseline and at months 24 and 48 in the four groups of patients enrolled in the study.

In the group that remained on initial treatment, the mean standard deviation (SD) number of new CLs at 48 months was 1.4 ± 1.0 (range 0–5) in the sc IFN *β*-1a group (45 patients), compared with 2.3 ± 1.3 (range 0–6) in the im IFN *β*-1a group (41 patients) and 2.2 ± 1.5 (range 0–7) in the glatiramer acetate group (43 patients). The mean number of new CLs was significantly lower in the sc IFN *β*-1a group than for either of the other treatments (*P* = 0.004 versus im IFN *β*-1a; *P* = 0.030 versus glatiramer acetate).  Figure 2 shows the mean number of total CLs for each time point. In the patients who began treatment after the first 24 months, the mean counts of new CLs were 3.0 ± 1.6 at 24 months and 1.5 ± 0.5 at 48 months (*P* < 0.001), white matter lesion mean was 2.1 ± 1.9 and 1.0 ± 0.2 (*P* < 0.001) and Gd+ lesions were 1.9 ± 1.0 and 0.5 ± 0.4 (*P* < 0.001), respectively. Mean annualized relapse rate was also significantly lower during treatment than pretreatment (*P* = 0.002). At month 48, the mean count of new white matter lesions and EDSS change were significantly different between sc IFN *β*-1a, im IFN *β*-1a, and glatiramer acetate, with more pronounced effects seen with sc IFN *β*-1a (*P* = 0.01 versus im IFN *β*-1a; *P* = 0.02 versus glatiramer acetate). The mean number of relapses did not differ between treatment groups.

### 3.3. Effect of DMD Therapy on Cortical Atrophy Progression

Mean baseline gray matter fraction was comparable across all the groups that retained the original therapy: 37.2% (sc IFN *β*-1a), 37.1% (im IFN *β*-1a), and 37.4% (glatiramer acetate). Compared to baseline, at month 48, the mean ± SD percentage decrease in gray matter fraction was 1.8% ± 0.6% in the 43 patients who were untreated for 24 months and then began active treatment and 1.4% ± 0.5% (*P* = 0.04) in the 129 patients who remained on active treatment for the duration of the study. Percentage change from baseline in gray matter fraction did not differ significantly among the treatment groups.

## 4. Discussion

The initial 24-month study showed the effect of DMDs in reducing the development of CLs in patients with RRMS [10], and the results presented here from the 24-month extension demonstrate that this effect is maintained over 48 months of treatment. Despite the low number of patients included in this study, the positive effect of DMDs was also observed in patients who commenced treatment after 24 months of no therapy. Moreover, this study indicates that high-dose, high-frequency sc IFN *β*-1a has a more rapid and pronounced effect on cortical pathology compared with both im IFN *β*-1a and glatiramer acetate.

Previous studies demonstrated that sc IFN *β*-1a significantly slowed progression of brain and gray matter atrophy in RRMS over 2 years [15, 16]. Our findings corroborate these observations. Indeed, all DMD treatments were found to reduce the accumulation of CLs, which correlates with the decline in cortical atrophy progression. It is likely that the effect of DMDs in reducing cortical atrophy may, at least partly, be the consequence of the downregulatory effect of DMDs on cortical inflammation. This is particularly relevant since there is a significant relationship between cortical pathology and some clinical aspects of MS, especially cognitive impairment [8, 9]. Increasing evidence suggests that cortical gray matter pathology, especially cortical atrophy, is strictly related to disability progression in MS [17]. We have previously observed that a low degree of cortical pathology was associated with the so-called “benign” course of MS [18], while the results of several independent studies converge in the suggestion that patients with a more severe physical and cognitive impairment are characterized by a more diffuse and marked gray matter atrophy, especially cortical thinning [19–22]. Finally, selective gray matter atrophy was relevant in patients with clinically isolated syndrome who converted early to MS [23].

Despite the limitations described previously [10], our study suggests that DMD-based therapy reduces the accumulation of inflammatory lesions not only in the white matter but also in the grey matter and supports the importance of an early disease-modifying treatment for RRMS.
